# The Value of the Cardiac Magnetic Resonance Intravoxel Incoherent Motion Technique in Evaluating Microcirculatory Dysfunction in Hypertrophic Cardiomyopathy

**DOI:** 10.1155/2023/4611602

**Published:** 2023-06-28

**Authors:** Alina Abulaiti, Quan Zhang, Haiyan Huang, Shuang Ding, Miriguli Shayiti, Shaoyu Wang, Yunling Wang, Wenxiao Jia

**Affiliations:** ^1^Department of Imaging, First Affiliated Hospital of Xinjiang Medical University, Urumqi 830011, China; ^2^MR Scientific Marketing, Siemens Healthineers, Shanghai 201318, China

## Abstract

**Objective:**

To evaluate the value of the cardiac magnetic resonance intravoxel incoherent motion (IVIM) technique in microcirculatory dysfunction in patients with hypertrophic cardiomyopathy (HCM).

**Methods:**

The medical records of 19 patients with HCM in our hospital from January 2020 to May 2021 were collected retrospectively, and 23 healthy people with a similar age and gender distribution to the patients with HCM were included as controls. All the included subjects underwent clinical assessment and cardiac magnetic resonance imaging. The original IVIM images were analysed, and the imaging parameters of each segment were measured. The HCM group was divided into non-hypertrophic myocardium and hypertrophic myocardium groups. The differences in imaging parameters between the normal and HCM groups were compared. A Spearman correlation analysis was used to explore the correlation between end-diastolic thickness (EDTH) and each IVIM parameter.

**Results:**

The D^*∗*^ and f values in the HCM group were lower than those in the normal group (*p* < 0.0001 and *p* = 0.004, respectively). The f, D, D^*∗*^, and EDTH values of the hypertrophic segment, non-hypertrophic segment, and normal groups were statistically significant (*p* < 0.05). The difference in D^*∗*^ values among the mild, moderate, severe, and very severe HCM groups was statistically significant (*p* < 0.05). There was a statistically significant difference in EDTH among the mild, moderate, severe, and very severe groups (*p* < 0.001). There were significant differences in the values of D, D^*∗*^, and f between the non-delayed enhancement group and the delayed enhancement group (*p* < 0.05). The EDTH values of 304 segments in the HCM group were negatively correlated with f (*r* = −0.219, *p* = 0.028) and D^*∗*^ values (*r* = −0.310, *p* < 0.001).

**Conclusion:**

The use of IVIM technology can achieve a non-invasive early quantitative assessment of microvascular disease in HCM without the injection of a contrast agent and provide a reference for the early diagnosis of and intervention in myocardial ischemia in patients with HCM.

## 1. Introduction

Hypertrophic cardiomyopathy (HCM) is a genetic disease characterised mainly by asymmetric thickening of the left ventricular wall caused by mutations in myocardial sarcomere-related genes [1]. Its pathological feature is myocardial cell hypertrophy, i.e., the disordered arrangement, interstitial fibrosis, and thickening of the small vessel wall in the myocardium [2], with an incidence of about 0.2% [3]. The main clinical features are dyspnoea, angina, and syncope. Some patients may have asymptomatic or initial symptoms of sudden cardiac death (SCD) caused by ventricular arrhythmia [4]. The pathological mechanism is myocardial fibrosis caused by myocardial ischemia. Coronary microvascular dysfunction (CMD) caused by an abnormal coronary microvascular structure and function is the main cause of myocardial ischemia, and its severity is closely related to ventricular hypertrophy and fibrosis. It provides strong evidence for the occurrence of adverse left ventricular remodelling, systolic dysfunction, and malignant arrhythmia and is an independent predictor of deterioration or death [5]. Studies have shown that most patients with asymptomatic HCM have reversible perfusion defects. Accordingly, identifying the risk of heart failure or sudden death in such patients is the focus of clinical practice [6].

Cardiac magnetic resonance (CMR) imaging is the most sensitive and specific method for evaluating CMD together with the multi-parameter quantitative indicators provided by a variety of postprocessing software. It can simultaneously provide morphological, functional, metabolic, and even molecular information to achieve non-invasive, comprehensive one-stop-shop scanning. Intravoxel incoherent motion (IVIM) imaging technology is a new CMR technology that reflects the diffusion parameters of water molecules in tissues with a bi-exponential and multi-b-value model; it quantitatively describes the signal changes caused by microcirculatory disturbances and intuitively reflects the pathophysiological information of tissue perfusion [7]. The absence of a contrast medium throughout the imaging process prevents contrast-related allergic reactions and their possible renogenic fibrotic changes.

Therefore, in this study, IVIM imaging was performed on each segment of the left ventricular myocardium in patients with HCM. Multi-segment and multi-angle analyses were conducted to achieve accurate positioning, and a quantitative evaluation was performed to identify differences in IVIM parameters between the normal group and the HCM group, between hypertrophic and non-hypertrophic segments, and between delayed enhancement segments and non-delayed enhancement segments; this was to reflect the internal correlation between the microcirculatory disturbance of local myocardial segments in HCM, myocardial morphological changes, and myocardial fibrosis to improve the clinical diagnosis and risk assessment of HCM and better guide clinicians to clarify the treatment direction, measures, and prognosis analysis of patients with HCM (this study will further explore the value of 3.0 T CMR IVIM in evaluating microvascular disease (MVD) in patients with HCM).

## 2. Methods

### 2.1. Subjects

The medical records of 19 patients with HCM who underwent a 3.0 T CMR examination in our hospital from January 2020 to May 2021 were analysed retrospectively. The case diagnoses were made with reference to the European Society of Cardiology and the American Heart Association's guidelines (AHA's guidelines) for the diagnosis and management of HCM [8, 9].

The inclusion criteria were as follows: left ventricular end-diastolic thickness (EDTH) ≥15 mm, patients with a family history of HCM with left ventricular EDTH ≥13 mm, and no other cardiac diseases related to myocardial hypertrophy. Other causes were excluded when the left ventricular maximum wall thickness of children ≥ normal predictive value + 2 SD.

The exclusion criteria were as follows: patients with other diseases that may lead to ventricular hypertrophy (e.g., coronary heart disease, hypertension, myocarditis, aortic valve disease, invasive cardiomyopathy, renal insufficiency, and physiological hypertrophy caused by excessive exercise in athletes), patients with previous surgical history, and patients with low-quality CMR images.

Additionally, 23 healthy subjects of a similar age and gender distribution to the patients with HCM were included in the study as a normal control group. The healthy participants had no history of myocardial microcirculatory abnormalities, and their CMR examinations were negative. All patients with HCM and the healthy participants received a clinical evaluation and a CMR examination. None of the study subjects had contraindications to magnetic resonance imaging examination, and signed informed consent was obtained before the examination. This study was approved by the ethics committee of our hospital.

### 2.2. Imaging

#### 2.2.1. Cardiac IVIM Imaging Scanning

The study used a Skyra 3.0 T superconducting magnetic resonance imaging system (Siemens Medical Systems, Germany) with an 8-channel cardiac phase control coil, precordial electrocardiogram (ECG) gating, and respiratory gating technology. The balanced steady-state free precession sequence was used for four-chamber and short-axis film sequence scanning. The repetition time (TR) was 2.8∼3.0 ms, the echo time (TE) was 1.1∼1.5 ms, the field of view (FOV) was 370 × 320 mm, the layer thickness was 8 mm, and the flip angle was 60°–70°.

**Figure 1:**
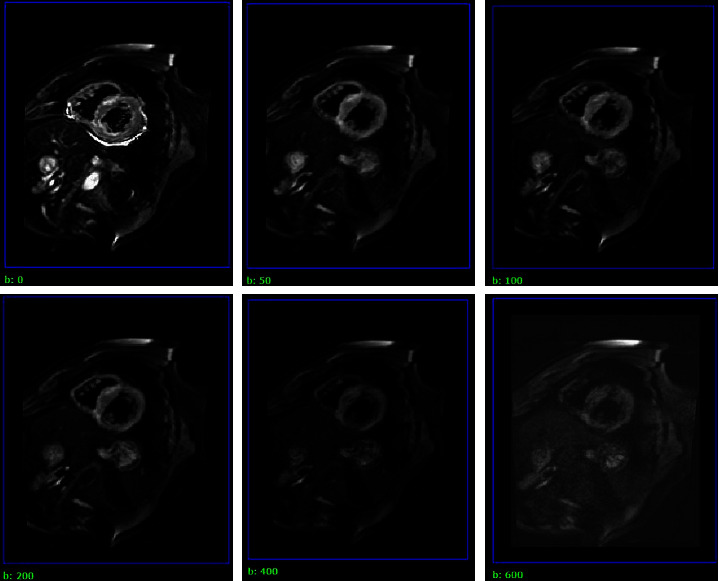
IVIM images with different b values in middle view of left ventricular short axis of HCM patients.

*Intravoxel Incoherent Motion Scanning.* On the four-chamber cine image, the time point with the minimum amplitude of myocardial motion in the middle and late diastole of the left ventricle was recorded as the trigger delay (TD) time of the IVIM sequence. Triple-slice images of the short-axis basal, middle, and apical segments of the left ventricle were acquired for different b values (0, 50, 100, 200, 400, and 600 s/mm^2^) (Figure 1).

#### 2.2.2. Cardiac Delayed Enhancement Scan

The following parameters were used: matrix = 84 × 128, TR was 300 ms, TE = 56.0 ms, layer thickness = 5.0 mm, layer spacing = 18 mm, FOV = 400 × 400 mm, and TD time = 350∼600 ms. For the end-expiratory occlusion scanning, the scanning time was 16∼25 s, and the heart rate was about 55∼100 times/min. The phase-sensitive inversion recovery sequence was performed 10∼15 min after the intravenous injection of Gd-DTPA (0.2 mmol/kg). The range of film sequences was replicated during scanning to obtain late gadolinium enhancement (LGE) images.

### 2.3. Image Analysis

Two imaging physicians with years of diagnostic experience conducted quality screening for all the images included in the medical imaging information system.

#### 2.3.1. Myocardial Segmentation

According to the AHA's proposed 17 myocardial segments, the short axis was divided into three parts according to the spatial position (i.e., basal segment, middle segment, and apical segment), making a total of 17 segments. In this study, the apical boundary of the left ventricular short axis was unclear, and it was difficult to observe. Due to the large measurement error, it was not analysed. Therefore, only 16 segments were included.

#### 2.3.2. Myocardial Thickness

The EDTH of the wall of each myocardial segment was measured. According to the cutoff value of the diagnostic criteria (15 mm) and the wall thickness measured in the HCM group, the HCM group was divided into a non-hypertrophic myocardium group (EDTH <15 mm) and a hypertrophic myocardium group (EDTH ≥15 mm); then, according to the degree of hypertrophy, the hypertrophic group was assigned to a mild hypertrophy group (15.00∼19.99 mm), a moderate hypertrophy group (20.00∼24.99 mm), a severe hypertrophy group (25.00∼29.99 mm), and a very severe hypertrophy group (≥30.00 mm).

#### 2.3.3. IVIM Imaging

The study used the commercial Medical Imaging Interaction Toolkit diffusion software to analyse and process the original images of each IVIM segment and measure the imaging parameters of each segment, including the slow apparent diffusion coefficient D (ADC_slow_), the fast apparent diffusion coefficient D^*∗*^ (ADC^fast^), and the fraction of ADC^fast^.

The measurement method was as follows. At the short-axis slice of the left ventricle, the region of interest (ROI) of each segment was drawn manually, and the average voxel size was about 60 mm^3^. During the measurement, the intracardiac blood pool and extracardiac fat components were avoided to be drawn into the ROI. The ROIs of the same segment, position, and size were measured three times, and the mean was calculated.

#### 2.3.4. Delayed Enhancement

According to the presence or absence of enhancement, they were divided into an enhanced group and a non-enhanced group.

### 2.4. Statistical Analysis

A statistical analysis was conducted using IBM SPSS 22.0 software. All measurement data were consistent with a normal distribution, which was expressed as $\bar{x}\pm s$. When the data were consistent with homogeneity of variance, an independent-sample *t*-test was used to compare two independent samples, and a least-squares difference *t*-test in a one-way analysis of variance was used to compare multiple groups. A non-parametric test was used when homogeneity of variance was not met, the Kruskal–Wallis test was used for comparisons among multiple sets of data, and the Mann–Whitney test was used for comparisons between groups. Spearman's correlation analysis was used to explore the correlation between two variables. A value of *p* < 0.05 was considered statistically significant.

## 3. Results

### 3.1. Basic Information and Clinical Characteristics

There was no significant difference in gender, age, height, weight, BMI, and heart rate between the HCM group and the normal group (Table 1). There were 368 myocardial segments in 23 normal subjects. The maximum EDTH was located in the basal and middle segments of the ventricular septum (segments 2, 3, 8, and 9). In the HCM group, there were 304 myocardial segments in 19 cases. There were 80 hypertrophic segments (26.32%) and 224 non-hypertrophic segments (73.68%). The maximum EDTH was also located in segments 2, 3, 8, and 9.

### 3.2. Overall Comparison of All Myocardial Segments between Normal Control and HCM Groups

**Figure 2:**
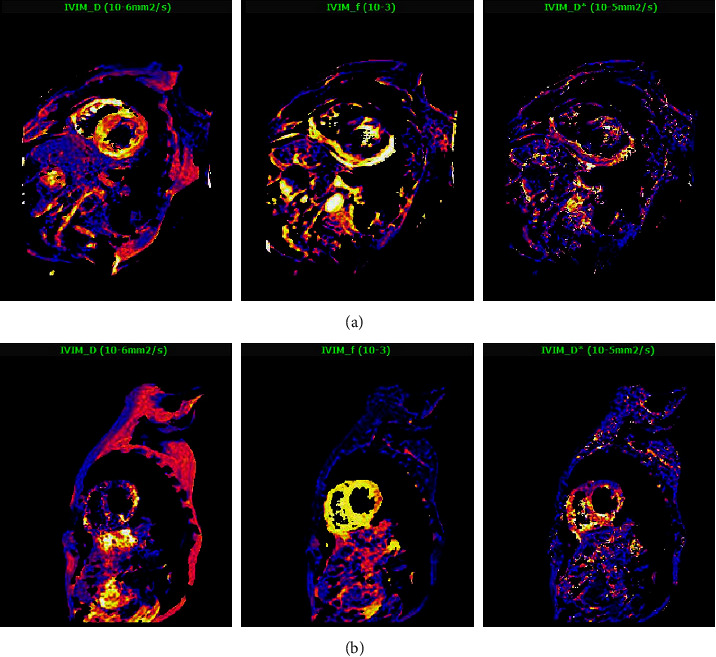
Short-axis IVIM images of HCM patients (a) and normal subjects (b).

The average EDTH value in the HCM group was 12.37 ± 3.67 mm, which was higher than that in the normal group (6.01 ± 1.57 mm), and the difference was statistically significant (*t* = 12.197, *p* < 0.001). The D^*∗*^ and f values of the HCM group were significantly different between the two groups (*t* = 10.371, *p* < 0.0001; *t* = 8.126, *p*=0.004, respectively). The D^*∗*^ and f values of the HCM group were lower than those of the normal group (53.17 ± 21.24 vs. 72.16 ± 17.73; 0.64 ± 0.27 vs. 0.87 ± 0.31, respectively). There was no significant difference in the D value between the two groups (*p*=0.164) (see Table 2 and Figure 2).

### 3.3. Comparison of the Non-Hypertrophic Segment Group, Hypertrophic Segment Group, and Normal Group within the HCM Group

The HCM group was divided into hypertrophic and non-hypertrophic segments according to 16 segments. The F, D, D^*∗*^, and EDTH values of the hypertrophic segment group, the non-hypertrophic segment group, and the normal group were statistically significant (*F* = 9.655, *p*=0.001; *F* = 5.314, *p*=0.041; *F* = 12.912, *p* < 0.001, respectively). In a pairwise comparison, D, D^*∗*^, and f in the normal group were higher than those in the hypertrophic segment, and the EDTH was lower than that in the hypertrophic segment (*p* < 0.05). The D^*∗*^ value in the normal group was higher than that in the non-hypertrophic group (*p* < 0.05), and the EDTH was lower than that in the non-hypertrophic group (*p* < 0.05) (see Table 3).

### 3.4. Comparison among Subgroups with Different Degrees of Hypertrophy in Patients with HCM

In the HCM group, 80 hypertrophic segments were divided into four groups according to the degree of hypertrophy: mild, moderate, severe, or very severe. There were 54 segments in the mild group (67.50%), 16 segments in the moderate group (20.00%), 5 segments in the severe group (6.25%), and five segments in the very severe group (6.25%). The difference in D^*∗*^ values among the mild, moderate, severe, and very severe HCM groups was statistically significant (*F* = 8.841, *p* < 0.001), while the difference in the f and D values among the groups was not statistically significant. There was a statistically significant difference in EDTH values among the mild, moderate, severe, and very severe groups (*F* = 17.192, *p* < 0.001), and the overall trends were upward (see Table 4).

### 3.5. Comparison between Non-Delayed Enhancement Group and Delayed Enhancement Group in the Normal and HCM Groups

In the HCM group, there were 17 cases of LGE delayed enhancement for the myocardium (89.47%) and 2 cases without delayed enhancement (10.53%). There were 88 enhanced segments (28.94%) and 216 non-enhanced segments (71.06%). The delayed enhancement of HCM was distributed mainly in the interventricular septum (segments 2, 3, 8, and 9). Delayed enhancement typically manifested as multiple patches and dots.

According to the 16 segments with or without delayed enhancement, HCM was divided into the non-delayed enhancement segment group and the delayed enhancement segment group. The statistical results showed that the values of D, D^*∗*^, and f between the delayed enhancement group, the non-delayed enhancement group, and the normal group were significantly different (*F* = 3.481, *p*=0.035; *F* = 11.621, *p* < 0.001; *F* = 6.655, *p* < 0.006, respectively). The difference in EDTH among the three groups was statistically significant (*F* = 9.512, *p* < 0.001). The largest EDTH was in the delayed enhancement group, while the smallest was in the normal group (14.37 ± 4.82 vs. 8.17 ± 3.14 vs. 6.01 ± 1.57, respectively) (see Table 5).

### 3.6. Correlation Analysis between EDTH and IVIM Parameters

The EDTH values of 304 segments in the HCM group were negatively correlated with the f (*r* = −0.219, *p*=0.028) and D^*∗*^ values (*r* = −0.310, *p* < 0.001), but they were not correlated with the D value.

## 4. Discussion

Hypertrophic cardiomyopathy cardiac imaging remains the basis for clinical diagnosis and management guidance; its main imaging manifestations are wall thickening (especially segmental or extensive hypertrophy of the ventricular septum or/and local wall) and irregular ventricular deformation/narrowing with or without varying degrees of left ventricular outflow tract stenosis. Although there are many invasive and non-invasive methods to evaluate CMD, CMR has a unique diagnostic value compared with many examination methods. Its one-stop examination can simultaneously evaluate cardiac morphology and function along with hemodynamics and myocardial tissue characteristics, and its non-invasive, non-radiative, multi-sequence, and multi-parameter characteristics overcome many of the limitations of other examinations, providing obvious advantages for evaluating CMD [10].

Among the various CMR technologies, as a new technology reflecting the molecular-level pathophysiology of myocardial tissue, IVIM imaging has important clinical significance for the diagnosis and treatment of cardiac diseases. The diffusion coefficient D reflects the diffusion movement of pure water molecules in voxels, i.e., tissue diffusion. Perfusion fraction f reflects the proportion of intravoxel microcirculation perfusion effect in the overall diffusion effect and can measure tissue blood volume. The pseudo-diffusion coefficient D^*∗*^ reflects the intravoxel microcirculation perfusion-related diffusion movement, allowing the blood flow in the tissue to be measured. The b value represents the diffusion-sensitive gradient factor. Due to the different molecular velocities and spatial distributions, parameter information with a low b value mainly reflects the perfusion effect of microcirculation, while a signal with a high b value mainly reflects the diffusion effect [11]. Setting the b value is critical in IVIM imaging, but there is no uniform standard for its number and size. When a low b value is set, the perfusion component increases, and the collected signal can fully describe the perfusion information of the tissue [12]. As the b value increases, the signal-to-noise ratio of the image is significantly reduced, there is a greater degree of myocardial signal attenuation, and the repeatability and accuracy of the measurement parameters are poor. During cardiac imaging, both high and low b values should be included to cover the information on water molecule diffusion and myocardial microcirculation perfusion. Although, in theory, the higher the b value, the more accurate the data fitting result and the better the image quality, multi-b value scanning prolongs the image acquisition time, during which patients can produce motion artefacts due to intolerance. Therefore, selecting too many b values is not appropriate for clinical application. After comprehensive consideration, six b values of 0, 50, 100, 200, 400, and 600 s/mm^2^ were selected in this study.

Another key factor for the success of IVIM scanning is the TD of ECG gating [13], which mainly solves the problem of signal loss by determining the minimum time window of cardiac motion.

Mou et al. [14] found that the left ventricular myocardium of patients with HCM was significantly lower than the D^*∗*^ myocardium of healthy people, indicating that the myocardium of HCM patients had low perfusion injury, which suggested that IVIM imaging was helpful for the prediction and monitoring of myocardial microcirculation. Xiang et al. [15] confirmed different myocardial perfusion parameters in different coronary artery blood supply areas via IVIM imaging. Finally, An et al. [16] showed that the D^*∗*^ and f values decreased significantly in the myocardial infarction area, while the D value showed a downward trend after PCI.

In the present study, the wall thickness of all segments in 17 patients with HCM was observed and compared. It was found that the wall thickness of 80 (26.32%) segments was ≥15 mm, and the thickest part was often located in the basal and middle segments of the ventricular septum (segments 2, 3, 8, and 9), which was consistent with other results [14]. At the same time, the average EDTH of the 16 segments in the HCM group was significantly higher than that in the corresponding segments in the normal control group, indicating that although HCM myocardial hypertrophy is most commonly located in the ventricular septum, the thickness of the segmental myocardium also changes accordingly, reflecting its asymmetry. At the same time, in this study, about 17 patients (89.47%) in the HCM group exhibited delayed enhancement, of which 88 segments (29.94%) were enhanced. Delayed enhancement generally manifested as multiple patches and dots, while a few patients showed diffuse distribution.

Compared with the normal group, the D^*∗*^ value of the HCM group was significantly decreased, proving that the perfusion of the HCM group was reduced, and there was microcirculatory disturbance. Then, the IVIM parameters of the normal group, the non-hypertrophic segments, and the hypertrophic segments were further analysed and compared; it was found that the f, D, and D^*∗*^ values of the hypertrophic segments and the non-hypertrophic segments were lower than those of the normal group (*p* < 0.05). The results revealed that the microcirculatory disturbance of HCM existed not only in the hypertrophic segment but also in the non-hypertrophic segment, and the degree of damage in the hypertrophic segment was more obvious. Compared with the normal group, the HCM non-enhanced group, and the enhanced group, it was found that the non-enhanced segment, like the enhanced segment, had reduced microcirculatory disturbance and perfusion, and the enhanced segment was more prominent. In other words, the degree of myocardial microcirculatory disturbance in the fibrosis area was heavier, and myocardial fibrosis was a risk factor for SCD. Therefore, microcirculation has an important prognostic value.

The results also showed that EDTH was negatively correlated with the f and D^*∗*^ values, indicating that the degree of microcirculatory injury was positively correlated with the degree of myocardial thickening of the left ventricular wall, i.e., the degree of myocardial thickening increased. In this experiment, among the three parameters (f, D, and D^*∗*^) of IVIM, the f and D^*∗*^ values well reflected the microcirculatory disturbance of patients with HCM, and the D^*∗*^ value was more sensitive than the f value.

Several studies have confirmed the correlation between CMD and poor prognosis. For instance, Ismail et al. [17] found that the apparent diffusion coefficient value could effectively evaluate the prognosis efficiency of HCM interstitial myocardial fibrosis. Villa AD et al.[18] detected the effects of local hypoperfusion and ischemia in patients with HCM by delayed enhancement and found that oedema or fibrosis caused by repeated microvascular ischemia was associated with an increased incidence of arrhythmia. Another study [19] showed that the perfusion damage in adjacent areas with delayed enhancement was more serious, suggesting that microvascular dysfunction played a promoting role in the generation of ischemia and alternative fibrosis, and severe microcirculatory damage would also affect the long-term prognosis of patients with HCM. Patients with CMD have significantly higher rates of cardiovascular events, including hospitalisation for heart failure, SCD, and myocardial infarction [20].

The results of the present study confirmed that there were differences in IVIM parameters between the HCM group without delay enhancement and the HCM group with delay enhancement. However, the LGE and extracellular volume (ECV) technologies described above require contrast agents, which cannot be used to examine patients with systemic lupus erythematosus or renal insufficiency. In contrast, IVIM does not require the injection of contrast agents to obtain relevant perfusion information. Compared with ECV, which reflects the deposition of intracellular and extracellular fibrosis, T1 mapping, which shows local myocardial fibrosis, and LGE, which shows the overall degree of myocardial fibrosis, IVIM can provide more information on myocardial tissue diffusion, including water molecule movement, microcirculation perfusion, and vascular reserve function between myocardial cells. Therefore, IVIM imaging technology provides a new perspective for future contrast-free CMR imaging and has the potential to provide prognostic value.

This study has some limitations. Due to the short research time and high cost of 3.0 T CMR examinations, only a small number of patients were included in the study. Additionally, since it was a single-centre study, there could have been some selection bias.

There are few studies on cardiac IVIM imaging, and there is no consensus on its imaging method and measurement standard; there is no clear reference value for the relevant parameters and no clear threshold reflecting the reduction in myocardial perfusion. The diagnostic efficacy of IVIM imaging for microcirculation perfusion abnormality and the prognostic value for adverse cardiovascular events are still unclear. Intravoxel incoherent motion imaging has certain requirements in terms of the general condition, respiratory control, and heart rate/rhythm of patients with cardiac diseases, and it also has high constraints for equipment performance and postprocessing software. Therefore, it has not been widely and fully applied in clinical practice for patients with HCM.

## 5. Conclusion

In summary, IVIM technology can achieve non-invasive early and quantitative assessment of MVD in HCM without the injection of a contrast agent and provide a reference for the early diagnosis of and intervention in myocardial ischemia in patients with HCM.

## Figures and Tables

**Table 1:** Basic information of HCM and normal group.

Group	Gender (male/female)	Age	Height	Weight	BMI	Heart rate
HCM	14/5	43.58 ± 11.67	165.87 ± 8.42	66.21 ± 11.27	24.31 ± 5.21	71.25 ± 7.31
Normal	16/7	41.75 ± 12.27	168.17 ± 9.72	65.37 ± 15.27	25.78 ± 4.38	73.14 ± 6.45
*X* ^2^/*t*	0.086	1.219	0.715	0.621	0.521	1.071
*p*	0.769	0.093	0.184	0.247	0.234	0.087

**Table 2:** Comparison of IVIM parameters between HCM group and normal group.

	HCM (*n* = 19)	Normal (*n* = 23)	*T*	*p*
D (*μ*m^2^/ms)	4.11 ± 3.43	4.29 ± 2.93	2.147	0.164
D^*∗*^(*μ*m^2^/ms)	53.17 ± 21.24	72.16 ± 17.73	10.371	<0.001
f (%)	0.64 ± 0.27	0.87 ± 0.31	8.126	0.004
EDTH (mm)	12.37 ± 3.67	6.01 ± 1.57	12.197	<0.001

**Table 3:** Comparison of parameters among normal group, non-hypertrophic segment group, and hypertrophic segment group.

	HCM		Normal	*F*	*p*
	Hypertrophic	Non-hypertrophic			
Number of segments	80	224	368		
D (*μ*m^2^/ms)	3.81 ± 2.97	4.16 ± 3.12	4.29 ± 2.93^*∗*^	5.314	0.041
D^*∗*^(*μ*m^2^/ms)	43.27 ± 17.39	57.98 ± 23.71	72.16 ± 17.73^*∗*^^#^	12.912	<0.001
f (%)	0.42 ± 0.31	0.76 ± 0.26	0.87 ± 0.31^*∗*^	9.655	0.001
EDTH (mm)	19.31 ± 4.12	8.17 ± 4.06	6.01 ± 1.57^*∗*^^#^	14.516	<0.001

^*∗*^The difference between the normal group and the hypertrophic segment was statistically significant. ^#^The difference between the normal group and the non-hypertrophic segment was statistically significant.

**Table 4:** Comparison of parameters between groups with different degrees of hypertrophy in HCM group.

	Mild	Moderate	Severe	Very severe	*F*	*p*
Number of segments	54	16	5	5		
D (*μ*m^2^/ms)	4.15 ± 3.75	4.38 ± 3.31	3.51 ± 1.37	4.81 ± 1.12	1.291	0.761
D^*∗*^ (*μ*m^2^/ms)	48.91 ± 31.91	36.19 ± 29.18	18.09 ± 4.12	21.87 ± 7.39	8.841	0.009
f (%)	0.53 ± 0.33	0.37 ± 0.38	0.59 ± 0.36	0.57 ± 0.22	2.065	0.081
EDTH (mm)	17.81 ± 4.16	24.86 ± 3.91	28.39 ± 0.86	34.17 ± 1.39	17.192	<0.001

**Table 5:** Comparison of parameters among normal group, non-delayed enhancement group, and delayed enhancement group.

	HCM		Normal	*F*	*p*
	Delayed enhancement	Non-delayed enhancement			
Number of segments	88	216	368		
D (*μ*m^2^/ms)	4.21 ± 3.79	3.86 ± 2.12^*∗*^	4.29 ± 2.93^*∗*^	3.481	0.035
D^*∗*^(*μ*m^2^/ms)	50.51 ± 21.43	59.59 ± 25.97^*∗*^^#^	72.16 ± 17.73^*∗*^	11.621	<0.001
f (%)	0.58 ± 0.35	0.79 ± 0.34^*∗*^^#^	0.87 ± 0.31^*∗*^	6.655	0.006
EDTH (mm)	14.37 ± 4.82	8.17 ± 3.14^*∗*^^#^	6.01 ± 1.57^*∗*^	9.512	<0.001

^*∗*^There was statistically significant difference compared with the delayed enhancement group. ^#^There was statistically significant difference between the non-delayed enhancement group and the normal group.

## Data Availability

All data generated or analysed during this study are included within the article.

## References

[1] Yin L., Xu H. Y., Zheng S. S. (2017). 3.0 T magnetic resonance myocardial perfusion imaging for semi-quantitative evaluation of coronary microvascular dysfunction in hypertrophic cardiomyopathy. *The International Journal of Cardiovascular Imaging*.

[2] Firth J., Firth J. (2019). Cardiology: hypertrophic cardiomyopathy. *Clinical Medicine*.

[3] Marian A. J., Braunwald E. (2017). Hypertrophic cardiomyopathy: genetics, pathogenesis, clinical manifestations, diagnosis, and therapy. *Circulation Research*.

[4] Maron B. J. (2018). Clinical course and management of hypertrophic cardiomyopathy. *New England Journal of Medicine*.

[5] Aguiar Rosa S., Rocha Lopes L., Fiarresga A., Ferreira R. C., Mota Carmo M. (2021). Coronary microvascular dysfunction in hypertrophic cardiomyopathy: pathophysiology, assessment, and clinical impact. *Microcirculation*.

[6] Karamitsos T. D., Arnold J. R., Pegg T. J. (2012). Patients with syndrome X have normal transmural myocardial perfusion and oxygenation: a 3-T cardiovascular magnetic resonance imaging study. *Circulation: Cardiovascular Imaging*.

[7] Spinner G. R., von Deuster C., Tezcan K. C., Stoeck C. T., Kozerke S. (2017). Bayesian intravoxel incoherent motion parameter mapping in the human heart. *Journal of Cardiovascular Magnetic Resonance*.

[8] Elliott P. M., Anastasakis A., Borger M. A. (2014). 2014 ESC guidelines on diagnosis and management of hypertrophic cardiomyopathy: the task force for the diagnosis and management of hypertrophic cardiomyopathy of the European society of Cardiology (ESC). *European Heart Journal*.

[9] Ommen S. R., Mital S., Burke M. A. (2020). 2020 AHA/ACC guideline for the diagnosis and treatment of patients with hypertrophic cardiomyopathy: executive summary: a report of the American college of Cardiology/American heart association joint committee on clinical practice guidelines. *Circulation*.

[10] Schindler T. H., Dilsizian V. (2020). Coronary microvascular dysfunction: clinical considerations and noninvasive diagnosis. *Journal of the American College of Cardiology: Cardiovascular Imaging*.

[11] Le Bihan D. (2019). What can we see with IVIM MRI?. *NeuroImage*.

[12] Hernandez L. E. (2018). Myocardial stress perfusion magnetic resonance in children with hypertrophic cardiomyopathy. *Cardiology in the Young*.

[13] Moulin K., Croisille P., Feiweier T. (2016). In vivo free-breathing DTI and IVIM of the whole human heart using a real-time slice-followed SE-EPI navigator-based sequence: a reproducibility study in healthy volunteers. *Magnetic Resonance in Medicine*.

[14] Mou A., Zhang C., Li M. (2017). Evaluation of myocardial microcirculation using intravoxel incoherent motion imaging. *Journal of Magnetic Resonance Imaging*.

[15] Xiang S. F., Zhang X. Q., Yang S. J. (2018). STROBE-A preliminary investigation of IVIM-DWI in cardiac imaging. *Medicine (Baltimore)*.

[16] An D. A., Chen B. H., Rui W. (2018). Diagnostic performance of intravoxel incoherent motion diffusion-weighted imaging in the assessment of the dynamic status of myocardial perfusion. *Journal of Magnetic Resonance Imaging*.

[17] Ismail T. F., Jabbour A., Gulati A. (2014). Role of late gadolinium enhancement cardiovascular magnetic resonance in the risk stratification of hypertrophic cardiomyopathy. *Heart*.

[18] Camaioni C., Knott K. D., Augusto J. B. (2020). Inline perfusion mapping provides insights into the disease mechanism in hypertrophic cardiomyopathy. *Heart*.

[19] Cecchi F., Sgalambro A., Baldi M. (2009). Microvascular dysfunction, myocardial ischemia, and progression to heart failure in patients with hypertrophic cardiomyopathy. *Journal of Cardiovascular Translational Research*.

[20] Ayub M. T., Kalra D. (2020). Coronary microvascular dysfunction and the role of noninvasive cardiovascular imaging. *Diagnostics*.

